# Clinical-scale expansion of mesenchymal stromal cells: a large banking experience

**DOI:** 10.1186/s12967-016-0892-y

**Published:** 2016-05-20

**Authors:** Chantal Lechanteur, Alexandra Briquet, Olivier Giet, Olivier Delloye, Etienne Baudoux, Yves Beguin

**Affiliations:** Department of Hematology, Laboratory of Cell and Gene Therapy, CHU of Liège and University of Liège, CHU Sart-Tilman, 4000 Liège, Belgium; Department of Medicine, Division of Hematology, CHU and University of Liège, Liège, Belgium

**Keywords:** Banking, Clinical-grade, GMP, MSC, Mesenchymal stem cells

## Abstract

**Background:**

Mesenchymal stromal cells (MSC) are largely investigated in clinical trials aiming to control inappropriate immune reactions (GVHD, Crohn’s disease, solid organ transplantation). As the percentage of MSC precursors in bone marrow is very low, these must be expanded in vitro to obtain therapeutic cell doses. We describe here the constitution of an allogeneic human third-party MSC bank from screened healthy volunteer donors in compliance with quality specifications and ISCT-release criteria and report follow-up of different aspects of this activity since 2007.

**Methods:**

68 clinical-grade large-scale MSC cultures were completed and analyzed. The whole process was described, including volunteer donor screening, bone marrow collection, mononuclear cell isolation and expansion over 4 weeks, harvesting, cryopreservation, release, administration and quality controls of the cells (including microbiology, phenotype, and potency assays).

**Results:**

From 59 validated donors, 68 cultures were completed (mean of final yields: 886 × 10^6^ cells/culture) and a total of 464 MSC aliquots have been produced and stored in liquid nitrogen (mean of 132.8 × 10^6^ cells/bag). Each MSC batch underwent extensive testing to verify its conformity with EBMT and ISCT release criteria and was individually validated. As of June 1 2015, 314 bags have been released and infused to patients included in 6 different clinical protocols. All thawed MSC units satisfied to release criteria and no infusion-related toxicity was reported.

**Conclusion:**

In conclusion, despite low passage cultures, we have been able to create an allogeneic “off-the-shelf” MSC bank with a large number of frozen aliquots and report here an efficient clinical-grade MSC banking activity in place for more than 7 years. Our challenge now is to produce MSC in compliance with good manufacturing practices (GMP) as, in the meantime, MSC have become considered as advanced therapy medicinal products (ATMP). Another significant challenge remains the development of relevant potency assay.

## Background

Mesenchymal stromal cells (MSC) were identified more than three decades ago by Friedenstein et al. as the stromal cells of the marrow microenvironment that support hematopoiesis [1]. MSC are multipotent progenitors capable of differentiating into various cells and tissues, such as chondrocytes, osteoblasts and adipocytes [2, 3].

In addition to multilineage differentiation and participation in the hematopoietic niche, MSC exert powerful immunomodulatory effects that include: inhibition of proliferation and function of T and B cells, inhibition of dendritic cell maturation and function and immune modulation of other immune cells such as natural killer (NK) cells and macrophages [4–6]. Due to the absence of co-stimulatory molecules and low HLA class I expression, MSC are not thought to be prone to immune rejection [7, 8]. This makes them ideal candidates for allogeneic use in both regenerative medicine and clinical applications in immune diseases such as graft-versus-host disease (GVHD), Crohn’s disease (CD), rheumatoid arthritis (RA) among others [9].

The International Society for Cellular Therapy (ISCT) has established the minimum criteria to define MSC and described them as a heterogeneous population of spindle-shaped, plastic-adherent cells isolated from bone marrow, adipose tissue, and many other tissue sources such as umbilical cord or cord blood. They must express certain cell surface markers (CD73, CD90, CD105) and lack expression of others (CD45, CD34, CD14, HLA-DR) and have the capacity to differentiate into osteoblasts, adipocytes and chondroblasts when cultured in particular in vitro conditions [10, 11]. Prior to clinical application, MSC must be significantly expanded to obtain therapeutic cell doses. Traditionally, MSC are obtained by ex vivo culture of the adherent cell fraction of bone marrow aspirates. The percentage of MSC among marrow cells is very low (0.01 to 0.001 % depending on age) but they can be easily isolated and expanded to reach adequate numbers for therapeutic doses. For this reason, cell processing facilities have established procedures for large scale production of MSC.

In the Laboratory of Cell and Gene Therapy (LTCG, CHU of Liège), we started in late 2006 a “MSC bank” based on clinical-grade expansion of MSC from BM samples obtained from healthy volunteer donors. Cells are produced according to the European Group for Blood and Marrow Transplantation (EBMT) consortium recommendations for defining common procedures for MSC isolation and expansion, as well as common release criteria, enabling multicenter trials with comparable MSC products [12]. Our first pilot clinical protocol (20 patients) evaluated the safety and preliminary efficacy of MSC to prevent graft rejection and GVHD after allogeneic hematopoietic cell transplantation (HCT) with non-myeloablative conditioning (NMHCT) [13]. In this study, HLA-mismatched NMHCT with MSC co-infusion appeared to be safe. Also, the prevention of death from GVHD and preservation of GVT effects suggested by this study are currently under investigation in a multicenter randomized study of MSC co-transplantation in patients given HLA-mismatched PBSC after non-myeloablative conditioning (NCT01045382).

We are currently involved in six clinical trials of MSC infusion in different settings including HSC transplantation (HCT) with myeloablative or non-myeloablative conditioning, cord blood transplantation (CBT), solid organ transplantation and severe or refractory autoimmune disorders such as Crohn’s disease. According to each protocol, the MSC dose varies from 1 to 4 × 10^6^ MSC/kg per infusion.

In this paper, we demonstrate the feasibility and describe the difficulties of setting up a large MSC bank from allogeneic donors for use in academic clinical trials. The whole process is described, including volunteer donor screening, bone marrow collection, mononuclear cell isolation and expansion over 4 weeks, harvesting, cryopreservation, release, administration and quality controls of the cells. The results of the 68 clinical-grade MSC cultures completed in 7 years of activity are also summarized. Finally, future challenges in MSC banking are also discussed and particularly the translation of the clinical-grade MSC production to a good manufacturing practice-compliant (GMP) process.

## Methods

### Allogeneic donor recruitment

The study was approved by the human and animal Ethics Committees of the University of Liege. Written informed consent was obtained from all bone marrow donors in accordance with the Declaration of Helsinki.

Allogeneic volunteer MSC donors were recruited among medical school students and hospital personnel and bone marrow samples collected exclusively at the CHU of Liège. The donor had to fill in a questionnaire to identify potential risks of disease transmission and the answers were analyzed according to a standardized interpretation checklist. The donor was then examined by a Senior Hematologist and a series of blood tests were obtained in hematology, general chemistry and serology (HBS Ag, HBC Ab, HIV Ab, HCV Ab, Syphilis + Nucleic Acid Testing for HIV-HBV-HIV). MSC donor eligibility criteria are similar to those of HSC donors but applied in a more restrictive fashion. For example, urgent medical need (UMN) was never considered for donor eligibility and virtually all donor non-conformities are considered as absolute contraindications. In some clinical trials, additional donor eligibility criteria may be applied. If found eligible, the donor had to sign an informed consent form and the marrow collection was scheduled within 30 days of the screening visit using standardized prescription form.

### Bone marrow collection

Briefly, bone marrow (50 ± 10 ml) was collected from the posterior iliac crest under local anesthesia in sterile conditions into sterile heparin-containing syringes. The physician completed a standardized bone marrow collection report. Then, syringes were immediately transported at room temperature to the LTCG where they were received by trained cell processing laboratory staff. At the time of reception, each syringe was inspected and its appearance, identifiers and time of reception was documented before being processed within 30 min. We always worked with fresh, never with frozen, bone marrow.

### Initiation of in vitro MSC cultures (Fig. 1)

MSC expansion cultures and quality controls were carried out as described in relevant SOP of the Laboratory of Cell and Gene Therapy (LTCG) at the CHU of Liege. Bone marrow was first transferred under laminar air flow in sterile tubes and a sample taken for analysis before dilution 1:1 with PBS buffer (Miltenyi Biotec). Isolation of mononuclear cells (MNC) from diluted bone marrow was obtained by Ficoll^R^ density gradient (GE Healthcare, Amersham Biosciences, Upsala, Sweden) and centrifugation at 450 g during 20 min. Since 2013, the manual procedure has been changed for an automatized and closed Ficoll procedure using the Sepax^R^ device (Biosafe, Eysins, Switzerland). Implementation of the Sepax^R^ system was performed after formal validation of the method according to the results of three separate experiments initiated to compare manual versus automated Ficoll mononuclear cell isolation. MSC produced from MNC obtained by both methods satisfied to their specifications. The Sepax system was implemented because it was safer (closed system) and MNC contained more CFU-F, leading to more performant cultures.

**Fig. 1:**
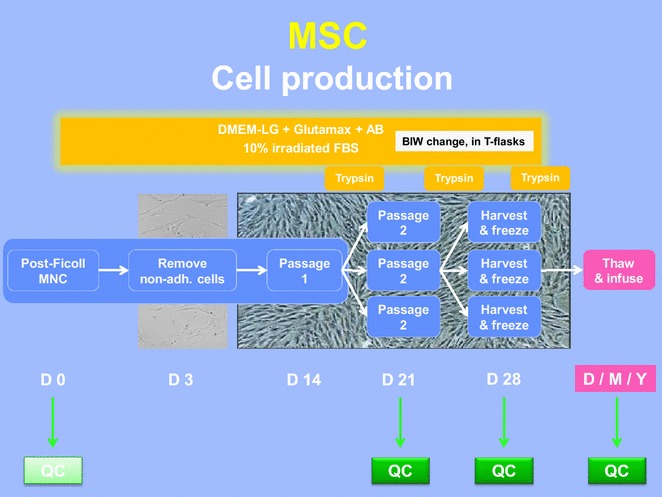
MSC clinical expansion. From ±50 mL of initial fresh BM, MNC cells were isolated by manual or automated ficoll isolation and seeded in T175 flasks. After a P0 expansion of 14 days, cells were harvested and re-loaded in new flasks for P1 expansion. One week later, cells were harvested and re-loaded according to the same scheme for P2 expansion before a final harvest around day 28. Quality controls were performed at different stages of the process

After washing (PBS), 28 × 10^6^ cells were seeded per 175 cm^2^ sterile tissue culture flasks (Falcon^R^) in Dulbecco's Modified Eagles Medium–Low Glucose with Glutamax (DMEM-GLX, Fisher-Bioblock, Invitrogen, Merelbeke, Belgium) supplemented with 10 % gamma-irradiated Fetal Bovine Serum (FBS, Hyclone, Perbio Sciences, Utah USA) and antibiotics (Penicillin/Streptomycin (P/S), Lonza, Petit-Rechain, Belgium) in a final volume of 28 mL. The flasks were incubated in an incubator at 37 °C, 5 % CO_2_ and 90 ± 5 % humidity. Adherent precursors were selected by removing non-adherent cells after 3 days and MSC were then expanded by replacement of the media twice a week. Cultures were monitored by microscopic observation for morphology and bacterial or yeast contamination. After generally 2 weeks of culture, MSC colonies were confluent (±70 %) and had to be replated.

### MSC trypsinisation and replating (1st and 2nd passages, Fig. 1)

When MSC were nearly confluent (70 %), they were trypsinized and replated at a lower density (4000 cells/cm^2^) to allow further cell expansion (first passage). Briefly, cells were first washed twice with phosphate-buffered saline (PBS), before incubation with trypsin–EDTA (Fisher-Bioblock, Invitrogen, Merelbeke, Belgium) for 5 min at 37 °C. Then, they were collected and washed before cell counting (trypan blue dye exclusion) on a Neubauer cell counting chamber. After dilution at the appropriate density in the complete culture media (DMEM + FBS + P/S, see above), cells were replated in T flasks and incubated at 37 °C with medium refreshed after 3 days. One week later, cells had generally reached ±70 % confluence and were replated for the second time according to the same procedure (second passage). At that time, several quality controls were performed (see below).

### MSC harvest (Fig. 1)

After a total of 4 weeks of culture (around day 28), MSC were generally ready for harvesting. Indeed, 1 week after the second passage, the cells have normally reached again more than 70 % confluence. They were first washed twice with phosphate-buffered saline (PBS) before incubation with trypsin–EDTA a few minutes at 37 °C. Cells were then collected in a harvest medium (PBS/5 % FBS), washed and then resuspended in an administration buffer [PBS/2.5 % human serum albumin (HSA)]. MSC were then washed in administration buffer before cell count on a Neubauer cell counting chamber and diluted at 2 × 10^6^ cells/ml (in administration buffer) before freezing. At that time, several quality controls were performed (see below).

### MSC cryopreservation

The MSC suspension (2 × 10^6^ cells/ml administration buffer) was transferred in appropriate sterile freezing bags (cryoMACS^R^, Miltenyi Biotec GmbH, Bergisch Gladbach, Germany). Aliquots containing 10 × 10^6^ to 190 × 10^6^ cells were prepared depending on the number of harvested cells. Then, the freezing solution (40 % PBS + 40 % of a 20 % HSA solution + 20 % DMSO) was added v/v to the cell suspension under agitation at 4 °C and cryopreservation was carried out with an automated cryofreezer (Thermoforma, Thermo Electron, Ohio) according to the following program:

**Table:** 

Duration	60 min until −160 °C
Steps of the programme	
Step 1	4 °C during 10 min
Step 2	⇩ à 1.5 °C/min until −12 °C
Step 3	⇩ à 45 °C/min until −50 °C
Step 4	⇧ à 9 °C/min until −28 °C
Step 5	−20 °C during 5 min
Step 6	⇩ à 1 °C/min until −35 °C
Step 7	⇩ à 1.5 °C/min until −45 °C
Step 8	⇩ à 6 °C/min until −160 °C
Step 9	−160 °C during 99 min

Each bag was considered as a MSC unit and stored in the vapor phase of a liquid nitrogen tank until validation and release for clinical use.

### Placebo preparation

As placebos were required in the context of some randomized clinical trials, placebo batches were prepared (and frozen) according to the same procedure as the cell suspension (but without addition of cells). Briefly, the placebo preparation consisted in the administration buffer (PBS/2.5 % HSA) that is transferred in appropriate sterile bags (see above). The freezing solution (40 % PBS + 40 % HSA + 20 % DMSO) was added v/v to the placebo solution under agitation at 4 °C and cryopreservation was carried out with an automated cryofreezer (Thermoforma, Thermo Electron, Ohio). Placebo bags are then stored in the vapor phase of liquid nitrogen until clinical use. When a placebo is requested for injection, it is thawed, diluted with PBS and injected according to exactly the same procedure as for cell products (see below). The only quality controls performed on placebo consist in sterility testing before cryopreservation as well as upon thawing. Endotoxin and mycoplasma were not tested on placebo preparations.

### Quality controls and traceability (Table 1)

Donor recruitment, bone marrow collection, MSC expansion culture, freezing and quality controls were carried out as described in relevant SOPs of the LTCG. From the bone marrow collection, during all steps of culture and cryopreservation, samples and cell containers (T-flasks, freezing bags) are labeled according to ISBT standards ensuring sustained traceability of the cellular product.

**Table 1:** Quality controls performed at (a) the different steps of the culture and their impact on batch validation and quality controls performed at (b) the thawing of the cells and their impact on batch validation

Test	Culture step	Material	Method	Specifications	Release criteria for downstream manufacture
**(a) MSC quality controls**					
Cell count	P1, P2, Harvest	Cell suspension	Cell counting (Neubauer cell counting chamber)	None	Informative
Cell viability	P1, P2, Harvest	Cell suspension	Trypan blue exclusion (Neubauer cell counting chamber)	None	Informative
Morphology	P1, P2, Harvest	Cell suspension	Microscope observation	Fibroblastic	Release criteria for batch validation
Aspect	P1, P2, Harvest	Cell suspension	Microscope observation	Cell suspension without aggregates	Release criteria for batch validation (harvest only)
Sterility	P0, P2 and harvest	Culture supernatant	BactAlert^R^	Sterile	Release criteria for batch validation (P2 and harvest)
Mycoplasma	P2 and harvest	Culture supernatant	Biochemical test (detection of mycoplasma enzymes by bioluminescence)	Absence of mycoplasma	Release criteria for batch validation
Endotoxin	P2 and harvest	Culture supernatant	LAL (limulus amebocyte lysate) detection E.P. 2.6.14.	Endotoxin level <2.5 U.I./ml	Release criteria for batch validation
Cell identity and purity (phenotype)	P2 and harvest	Cell suspension	Facs analysis	CD90 > 70 % CD105 > 70 % CD73 > 70 % CD14 < 5 % CD34 < 5 % CD45 < 5 % CD3 < 1 %	Release criteria for batch validation
Karyotype	P2 and harvest	Cell culture	Chromosome analysis	Absence of abnormal chromosomal structure and/or number	Release criteria for batch validation
Immunosuppressive properties	Harvest	Cell culture	MLR with stimulated PBMC-cell cycle analysis	>25 % Inhibition of PBMC-induced proliferation (10/1 ratio)	Release criteria for batch validation
**(b) Post-thaw MSC quality controls**					
Cell count	Thawing	Cell suspension	Cell counting (Neubauer cell counting chamber)	Yes, cell number should be > × 10E6 and < × 10E6 cells/kg recipient According to the clinical protocol	Release criteria
Viability	Thawing	Cell suspension	Trypan blue exclusion (Neubauer cell counting chamber)	50 %	Release criteria
Sterility	Thawing	Thawed cell product	BactAlert^R^	Sterile	No, results are not known at the time of infusion. Cells are released according to sterility results at harvest

All components (equipment, starting cellular material, reagents, materials, personnel and methods) used in the manufacturing process were recorded.

At each step of the MSC expansion process, cells were submitted to quality controls (see below). Some of these were only informative while others were considered as release criteria according to the EBMT guidelines (Table 1).

We did not verify the absence of adventitious agents in FBS or Trypsin–EDTA. For this assessment, we relied on reagent certificates of analysis provided by the manufacturers.

### Phenotypic characterization of MCS

Analysis of cell-surface molecules was performed on MSC cultures using flow cytometry. Harvested cells were washed with PBS containing 5 % HSA. Around 2 × 10^5^ cells were resuspended in 90 μL PBS containing 5 % HSA, and incubated for 10 min on ice in the dark, with the following MAb: APC-conjugated CD73 (IgG1, AD2 clone), PE-conjugated CD105 (IgG1, 43A4E1 clone), FITC-conjugated CD90 (IgG1, DG3 clone), PerCP-conjugated HLADR (IgG2a, AC122 clone), vioblue-conjugated anti-CD45 (IgG2a, 5B1 clone), vioblue-conjugated anti-CD14 (IgG2a, TÜK4 clone), vioblue-conjugated anti-CD34 (IgG2a, AC136 clone), vioblue-conjugated anti-CD3 (IgG2a, BW264/56 clone). Cells incubated with their corresponding isotype control (IgG1 PE, FITC and APC: IS5-21F5 clone, IgG2a vioblue and PerCP: S43.10 clone Miltenyi Biotec) were also included. Data were acquired on a Macsquant Flow Cytometer (Miltenyi Biotec) by collecting a minimum of 10,000 events and analyzed with Macsquantify software.

### Mesenchymal stromal cells differentiation assays

Fat, bone and cartilage differentiation assays were carried out as described by Pittenger et al. [2] and revealed by staining with oil red O, alizarin red and toluidine blue, respectively. Differentiation media were home-made.

### MSC immunosuppression assays

1 × 10^4^ MSC were plated in triplicates in round-bottom 96-well plates (Becton–Dickinson) in a total volume of 100 μl of RPMI 1640 medium supplemented with 10 % FBS, 100 U/ml penicillin, 100 mg/ml streptomycin, l-glutamine (2 mM) (all from Lonza), sodium pyruvate (100 mM), non-essential amino acid (NEAA) (100 mM) and 5 × 10^−5^ M β-mercaptoethanol (β-ME) (all from Gibco, Merelbeek, Belgium). After 4-hour incubation, MSC were irradiated at 25 Gy using a 137Cs source (GammaCell 40, Nordion, Ontario, Canada).

Allogeneic human peripheral mononuclear cells (PBMC) were isolated from a blood sample (healthy volunteer donor) by Ficoll Paque^R^ Plus density gradient. PBMC (5 × 10^4^ or 1 × 10^5^) were then added to wells in a total volume of 200 μl containing or not irradiated MSC, in the presence of anti-αCD3/CD28 microbeads (Invitrogen, Dynal A/S, Oslo, Norway). Co-cultures without anti-αCD3/CD28 microbeads were used as controls. Cells were then incubated at 37 °C in 5 % humidified air for 4 days. Cell cycle analysis of PBMC stimulated or not with anti-αCD3/CD28 microbeads and cultivated during 4 days with or without MSC were performed using CycleTEST^R^ Plus DNA Reagent Kit (Becton–Dickinson). The percentage of cells in the different phases of the cell cycle was determined with the Macsquant Software (Miltenyi) or the Modfit Software (Becton–Dickinson). The effect of MSC on PBMC stimulation responses was calculated as percentage suppression compared with the proliferative response in the positive control without MSC (+- standard deviation of the mean). The positive control was set to 0 % suppression.

### Cytogenetics

Karyotyping was performed at the Genetics Department of the Hospital by the Q-banding technique and analyzed with Cytovision^R^ software.

### Microbiology testing

MSC sterility was assessed by bacterial culture (aerobia, anaerobia and fungi with Bactalert^R^; Microbiology department of the Hospital), mycoplasma screening (luminometry, Mycoalert^R^, Lonza; Microbiology department of the Hospital) and endotoxin detection (limulus test, European pharmacopeia 2.6.14, Pharmacy department of the Hospital).

### MSC release and thawing (Table 1b)

MSC are only prescribed and infused in the context of a clinical trial. Thus, patient eligibility/inclusion criteria, clinical evaluation and laboratory testing are protocol-specific and listed in each of the respective clinical trial descriptions. If the patient is eligible according to all protocol criteria, he must sign the study informed consent form to allow release of the cells (a frozen MSC product is released for clinical use specifically for a patient according to the prescribed MSC dose and the patient weight).

If MSC are delivered to a patient inside the hospital, they are thawed at the LTCG. Briefly, the MSC bag is protected in a sterile plastic bag and thawed in a 37 °C water bath for a few minutes. If the MSC bag is in a dual packaging (bags cryopreserved after April 2008), the bag is just immersed in the water bath a few seconds to take off easily the second packaging. Then the bag in its first packaging is quickly thawed in the water bath in a sterile protective bag. The bag is taken out of the water when the access sites are just thawed; there must still be small ice clots in the bag. Then, the bag is quickly transferred under the laminar flow and immediately perforated with a sampling-site coupler and diluted with a PBS buffer [1:0.75 (MSC solution:PBS)] (Clinical Grade, Miltenyi Biotec, Utrecht, The Netherlands) to avoid DMSO toxicity. Quality controls are performed after thawing of the cells (Table 1b). Numeration and viability are assessed by trypan blue coloration and cell count on a Neubauer cell counting chamber. We thus chose this method because it was easy and rapid (MSC must be infused as quickly as possible after thawing) and we did not have a flow cytometer available in the clean room to rapidly use other cell counting methods (7-AAD, PI). Besides, we do not routinely re-assess phenotype, mycoplasma and endotoxins after thawing. Indeed freezing/thawing steps have been validated with all critical QC parameters meeting eligibility criteria (Table 2c). In addition, as thawed cells must be infused as soon as possible, it is not possible to proceed with these QC before infusion. Moreover, the only new reagent introduced during the thawing step is PBS which is devoid of mycoplasma and endotoxins.

**Table 2:** MSC culture process validation

**(a) Cultures rates**					
	1st Exp.	2nd Exp.	3rd Exp.		
BM sample (mL)	28	52	55		
MNC on day 0	180 × 10^6^	195 × 10^6^	260 × 10^6^		
MSC on day 28	225 × 10^6^	165 × 10^6^	356 × 10^6^		

**(b) Phenotypes**					
Marker	Eligibility criteria (%)	1st Exp. (%)	2nd Exp. (%)	3rd Exp. (%)	Conformity
CD73	>70	96.9	99.9	99.4	Ok
CD105	>70	85.6	90.3	77.8	Ok
CD90	>70	100	98.2	100	Ok
CD34	<10	1.8	8.8	0.92	Ok
CD45	<10	1.1	1.10	0.55	Ok
HLA-DR	<10	0.84	0.63	0.52	Ok
CD80	<10	1.0	0.7	0.45	Ok
CD31	<10	1.0	0.9	0.62	Ok

**(c) Post-thaw**					
Test	Eligibility criteria	1st Exp.	2ndExp.	3rd Exp.	Conformity
Initial cell number	Na	100 × 10^6^	70 × 10^6^	55 × 10^6^	Na
Initial viability	Na	95.8 %	82.5 %	86.0 %	Na
Post-thaw cell count	Na	100 × 10^6^	56 × 10^6^	43 × 10^6^	Na
Post-thaw cell viability	>50 %	89.5 %	78.0 %	78.0 %	Ok
Post-thaw cell culture expansion	Na	Ok	Ok	Ok	Na
Sterility	Sterile	Ok	Ok	Ok	Ok
Mycoplasma	Absence of mycoplasma	Ok	Ok	Ok	Na
Endotoxin	Endotoxin level <2.5 U.I./ml	Ok	Ok	Ok	Na

Phenotype					
CD73	>70 %	99.5 %	96.1 %	87.9 %	Ok
CD105	>70 %	93.6 %	70.3 %	70.0 %	Ok
CD90	>70 %	100 %	99.6 %	99.8 %	Ok
CD34	<10 %	3.1 %	2.7 %	2.6 %	Ok
CD45	<10 %	1.0 %	1.9 %	1.0 %	Ok
HLA-DR	<10 %	1.0 %	1.2 %	1.0 %	Ok
CD80	<10 %	0.4 %	0.8 %	0.4 %	Ok
CD31	<10 %	0.6 %	1.2 %	0.6 %	Ok

Results of three large-scale MSC cultures for initial process validation: culture rates, phenotypes at final harvest and post-thaw parameters are reported

The cell product is then transferred in an appropriately labeled sterile transfer bag and transported to the hematology department of the hospital for infusion to the patient. The released MSC product can also be transferred frozen in a dry-shipper at −160 °C to another center for infusion after inclusion of a patient in a multicenter clinical protocol. Upon preparation before shipment, the bag canister, dry shipper and protective box are labeled as described in the transportation SOP. The shipper is placed in a protective box and transported by a specialized transporter ordered by the destination hospital. In the recipient tissue bank, cells are then thawed according to the LTCG standard procedure.

### Population doubling level (PDL) calculation

PDL was calculated according to the formula PDL = 3.322 (log Y−log I) where Y = number of cells harvested and I = number of cells inoculated at P1.

### Doubling time (DT) estimation

Doubling time (DT) was calculated according to the formula DT = *t* × log (2)/log (number of cells harvested/number of cells inoculated), where *t* is the time in hours between passage 1 and harvest of the cells.

### Statistics

Results are reported as mean ± standard error of the mean (SEM). Comparisons between conditions were made using Student unpaired *t* tests with GraphPad Prism 5.00 software (GraphPad Software, La Jolla, CA, USA).

## Results

### Large-scale cultures—process validation

After a few small-scale MSC expansions to set up the process, three large-scale clinical MSC cultures were initiated for validation with three different bone marrow (BM) samples obtained from healthy volunteer donors. All quality controls fulfilled pre-defined qualification criteria (Table 2).

Freezing/thawing steps were also validated. For each previous culture, frozen MSC were thawed and critical parameters were evaluated. Again, all quality controls met eligibility criteria (Table 2c). Thawed cells were also devoid of bacterial, mycoplasma and endotoxin contamination.

Shelf life determination of the product after thawing was also assessed. Cells from four different bags were left (or not = control) in the post-thaw buffer at room temperature for different times (1, 2, 4H). At each time point, cell count and viability were assessed. Viability and cell count seemed stable for 2 h but drop significantly when kept for longer periods. Cells were also seeded in culture flasks to test their proliferation potential. When replated, the cell proliferative potential was affected as early as after 1 h in the post-thaw buffer.

The effects of long-term cryopreservation were not systematically addressed. However, three MSC bags were thawed after 7–8 years of storage and showed excellent post-thaw viability (80, 77 and 83 %, respectively) and recovery (83, 71 and 94 %, respectively). This indicates a good stability of the cell product during long-term storage in liquid nitrogen. Systematic evaluation of long-term MSC stability is scheduled for future batches produced under GMP recommendations.

### MSC banking

After appropriate validation, we started in November 2006 a clinical-grade third party MSC bank following the EBMT manufacturing process.

During the past 7.5 years, 61 donors were screened. One donor was discarded for multiple allergies and collection was technically impossible for another one. From the 59 validated donors (36 females and 23 males), 70 large-scale MSC expansions were initiated and completed (Table 3). Donors were between 18 and 52 years of age (median 26.7 years). There is no standard for MSC donor age. Ten percent (7/68) of our donors were older than 40 years and 90 % were between 18 and 40, with a mean of 29 years. The older donors were collected at the beginning of our banking activity, but now we select donors younger than 40 years of age. Volumes of collected bone marrow ranged from 25 to 70 mL (median 50 mL) and total number of mononucleated cells obtained after ficoll ranged from 124 to 956 × 10^6^ (median 280 × 10^6^).

**Table 3:** MSC production between 2007 and 2015

Criteria	Min.	Max.	Median
MSC culture parameters			
Donor age (years)	18	52	26.7
BM sample volume (mL)	25	70	50
Mononuclear cells post-Ficoll (×10^6^)	124	956	280
CFU-F (number per 2 × 10^6^ cells)	2	96	25
Final MSC yields (×10^6^)	19	5431	546
PDL (P1-harvest)	2.38	7.18	4.69
DT in hours (P1-harvest)	46.8	141	71.7
Viability (%)	72	100	85
Number of aliquots (=bags)/culture	1	45	4
Number of cells/aliquot (×10^6^)	19	189	110

**Fig. 2:**
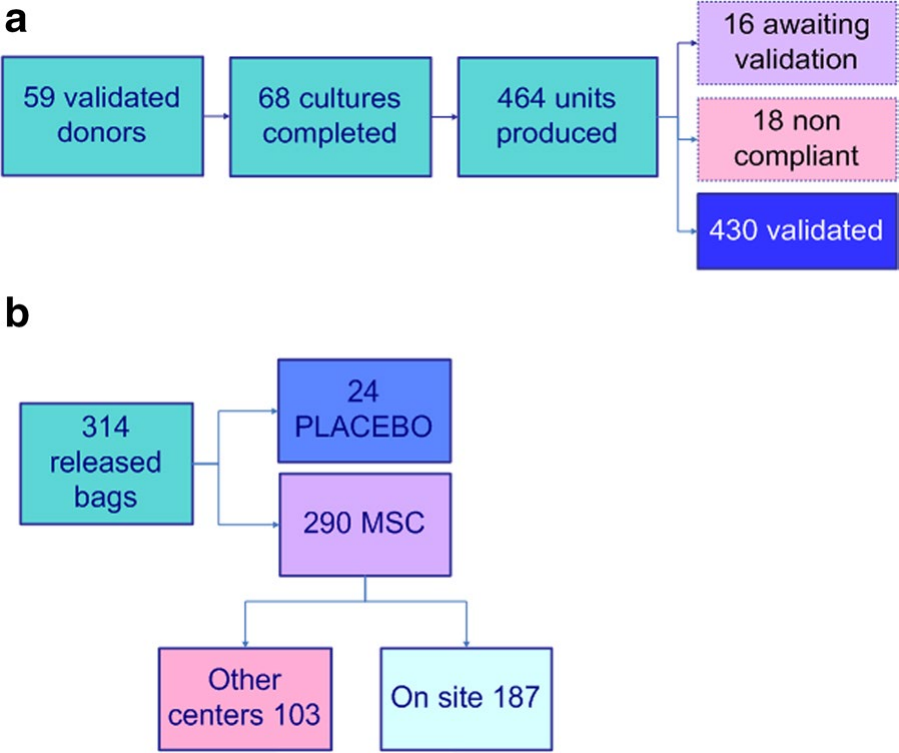
MSC banking: production and release activity. **a** Production: From 59 validated donors, 68 cultures were completed allowing freezing of 464 MSC bags. From these, 430 are already validated, 18 non-compliant and 16 are still awaiting validation. **b** Release: Since 2007, 314 bags have been released. From those, 290 were MSC and 24 placebo; 187 were released on site and 103 were sent to other Belgian centers

All the initiated cultures except two gave rise to colonies and completed the MSC culture process resulting in 1 to 45 MSC unit bags per culture. A total of 464 MSC bags have been produced and stored from the 68 completed cultures (Table 3; Fig. 2).

Final yields ranged from 19 × 10^6^ to 5431 × 10^6^ cells with a median of 546 × 10^6^ cells per culture. Population doubling levels (PDL) between passage 1 and passage 3 (harvest) ranged from 2.38 to 7.18 with a median of 4.69. Doubling times (DT) ranged from 46.8 to 141 h with a median of 71.7 h. We didn’t observe any correlation between age and Colony-Forming Units or between CFU-F and yield of the culture/mL BM processed (data not shown). However, although sex didn’t influence the yield of CFU-F (P = 0.55), male BM samples gave rise to significantly higher total cell yields compared to cultures obtained from female BM samples (P = 0.0011).

Viability at harvesting was generally excellent with a mean of 88.9 ± 5.5 %. All the harvested MSC were frozen in adequate aliquots at −160 °C in liquid nitrogen. Aliquots contained various numbers of cells in order to cover a wide range of patient weights, considering that the ideal MSC dose is ranging between 1–4 × 10^6^/kg recipients. MSC were stored in bags containing between 19 × 10^6^ and 189 × 10^6^ cells with a mean of 132.8 × 10^6^ cells per bag. We did not freeze standardized quantities because the required cell count for infusion is defined by the protocol in which the patient is included as a weight-based dose. The acceptable dose is always within a specified range, for example 1–2 or 3–4 × 10E6 cells/kg. The minimal cell count is the minimal acceptable dose of viable cells multiplied by the patient’s weight. So when a patient is included in a clinical trial, we choose an aliquot containing the optimal amount of cells according to the dose prescribed in the study and the patient weight. In this context, cell doses as small as 20 × 10E6 cells can be stored and released for low-weight patients (pediatric cases).

All cultures were initiated with fresh cells but due to technical limitations, a maximum of 300 T175 cm^2^ were replated at passage 2. If cell number exceeded this capacity, the cells were frozen at P2 and thawed later to allow further expansion and final harvest. Each harvest gave rise to one MSC batch so that one culture can generate one or more batches.

The large range in the final yields is due to variability in the intrinsic quality of the collected BM and also to variability in the efficiency of the different FBS batches (always pre-validated but some were better than others). As explained in the text, two cultures did not expand and were discarded. No T flask was discarded. Noteworthy, the extreme expansion rates (low and high) were rare, with most cultures (>80 %) yielding >200 × 10^6^ cells.

### Quality controls and compliance with release criteria

Each MSC batch underwent extensive testing to verify its conformity with release criteria and was individually validated. EBMT release criteria were applied to release batches but retrospective analysis revealed that cells were also compliant with the stricter ISCT phenotype criteria (>95 % expression CD90, CD105, CD73; <2 % for CD14, CD45, CD34 and <1 % for CD3). HLA-DR expression was also evaluated for all produced batches and was always below 2 % at harvest, but this was not a release criterion.

**Fig. 3:**
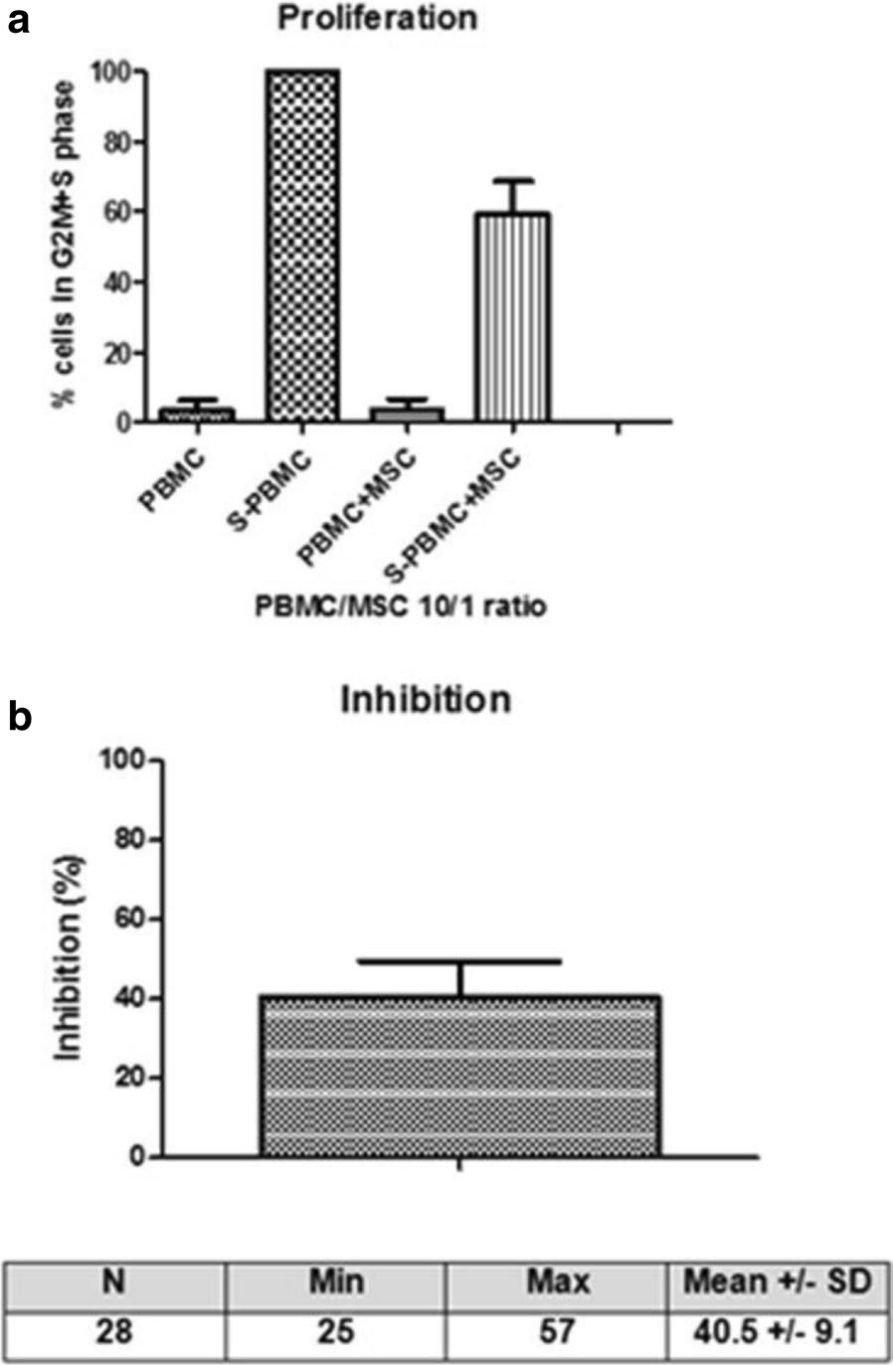
MSC immunosuppressive properties. Inhibition of PBMC proliferation by third party MSC: PBMC (100,000 or 50,000) were stimulated (S-PBMC) with anti-αCD3/CD28 microbeads during 4 days with or without irradiated (25 Gy) MSC (10/1 or 5/1 PBMC/MSC ratios) added at the beginning of the culture. Proliferation was assessed by analysis of the cell cycle by flow cytometry (N = 28). Result are expressed as the percentage of cells present in S + G2 M phases (**a**) and as the percentage of inhibition compared to the stimulated PBMC condition alone (**b**) for the 10/1 PBMC/MSC ratio

All but 3 batches satisfied to release criteria. Indeed, the batches from two cultures had to be discarded for non-conformity due to a donor constitutional anomaly detected by karyotypic analysis and further confirmed by analysis of donor lymphocytes. One batch was discarded due to a positive bacteriology result. MSC were also evaluated in a stimulation assay by incubation with anti-CD3/CD28. As shown in Fig. 3, proliferation of stimulated PBMC was significantly reduced by the addition of MSC [mean inhibition (10/1 ratio): 41 ± 9 %, range 25–57 %]. Thawed cells were cultured and also demonstrated good inhibitory properties (>25 % inhibition in a 10/1 ratio; data not shown). In summary, 464 MSC bags have been produced and frozen in our facility during the past 7 years (Fig. 2). Among them, 430 (93 %) have already been validated according to EBMT consortium release criteria and 16 are still awaiting validation. Eighteen bags had to be discarded (3.9 %) (Fig. 2).

All placebo bags were sterile and fulfilled release criteria.

### MSC release and thawing

All MSC products validated for clinical use were listed according to their cellular content, which enabled us to choose the adapted MSC product for each patient according to his/her weight and the dose specifications of the clinical trial in which the patient was included.

When a patient is included in a clinical trial, the selected MSC bag(s) is (are) released and thawed for infusion. Thawed MSC are quickly diluted with buffer and an aliquot is collected to assess MSC numeration and viability. If cell recovery is adequate for dose specifications and viability above 50 %, MSC infusion is allowed. Thawed cells must be infused as soon as possible and in all cases within 1 h after thawing. In order to respect the set time limit, before each scheduled infusion, we verify that the patient is in good condition and ready to receive the MSC before thawing the bag.

As of June 1 2015, 314 bags have been released (24 placebo and 290 MSC bags) and infused to patients. Among these, 103 were transported frozen to other Belgian centers before being thawed on site and 187 were prepared in the LTCG (Fig. 2b).

**Fig. 4:**
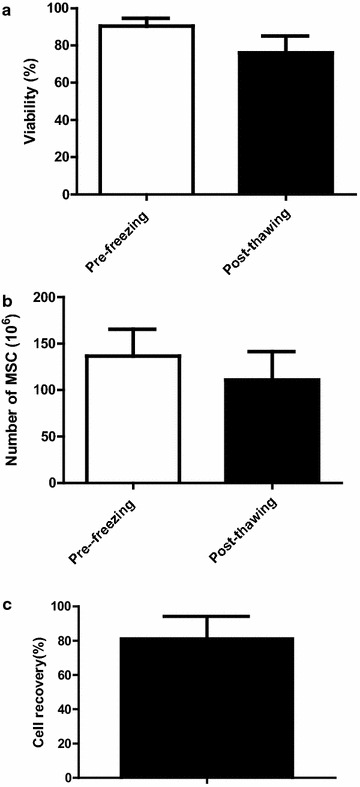
MSC post-thaw viability and cell recovery. Pre- and post-thaw MSC viability (**a**), MSC number (**b**), as well as recovery of cell number (**c**) (N = 170)

Mean viability at freezing was 90 ± 4 % (Fig. 4a), ranging from 80 to 100 %. Viability dropped to 76 ± 9 % after thawing (range 50 % to 96 %). The mean decrease of viability during thawing was 14 % (P < 0.0001). However, all thawed MSC units satisfied to release criteria as none showed viability below 50 %. To our knowledge, there is no standard in Europe for viability of such products and this 50 % viability cutoff was accepted by our regulatory authorities. However, the mean viability of our MSC after thawing was 76 ± 9 % (Fig. 4a) which is quite higher indeed. Noteworthy, very few bags showed viabilities between 50 and 60 % (3/167 = 1.8 %).

The mean cell content of the released bags was 136.6 ± 28.8 × 10^6^ cells (range 65 to 189 × 10^6^) before freezing (Fig. 4b) and 111 ± 30.5 × 10^6^ after thawing (range 45.5 to 189 × 10^6^), with a mean recovery of 81 ± 13 % (range 50 to 115 %) (Fig. 4c).

All thawed bags (MSC and Placebo) were subjected to bacteriological analysis and results were all negative.

### Post-thaw MSC potency

**Fig. 5:**
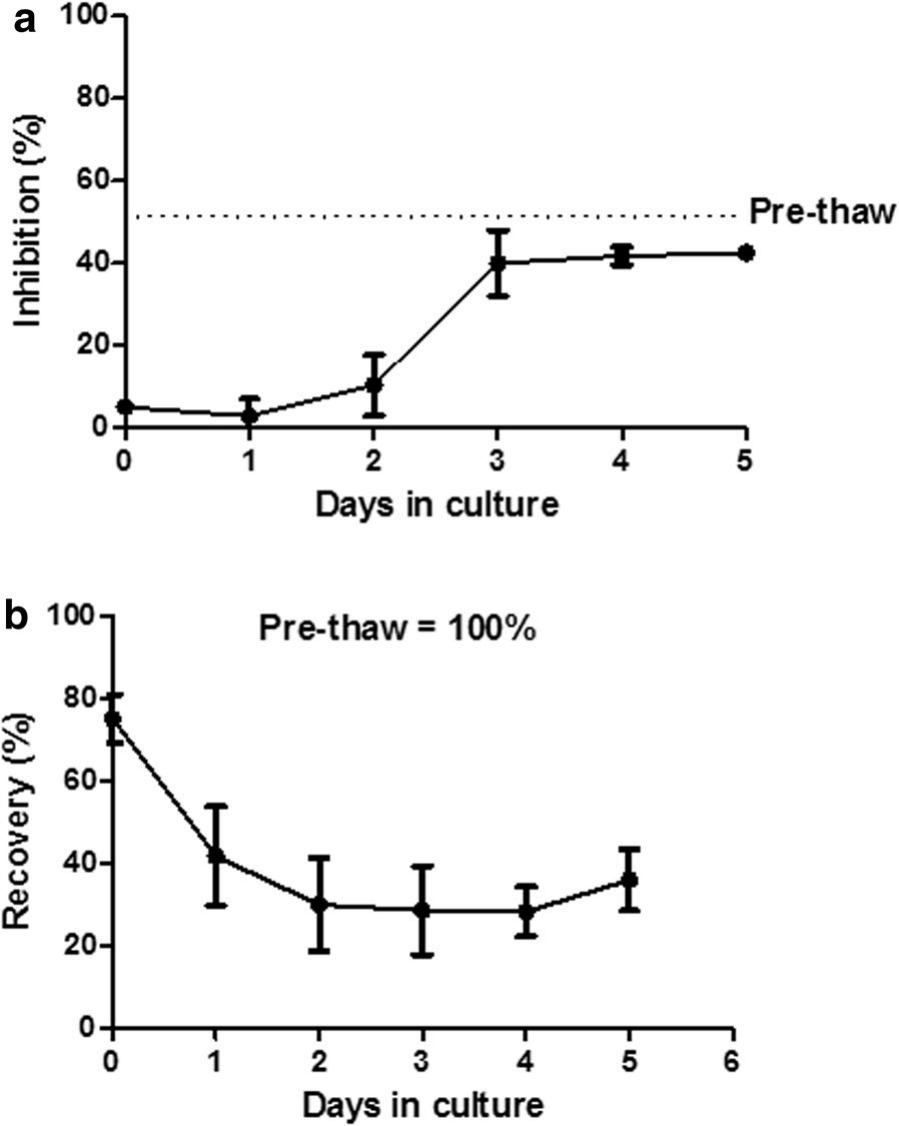
MSC post-thaw immunosuppressive properties. MSC bags were thawed (N = 5), washed and replated in flasks in complete media. Immunosuppressive properties (**a**) and cell recoveries (**b**) were evaluated at different time points after thawing

In order to evaluate post thaw MSC immunosuppressive properties, five MSC bags were thawed in separate experiments. Cells were washed in tubes and resuspended in complete media before being plated in T175 cm^2^ flasks. At different time points (day 1, 2, 3 and 4), cells were detached and evaluated for different parameters: recovery (compared to the number of seeded cells after washing), viability, phenotype, differentiation and immunosuppressive properties. Thawed MSC demonstrated very poor PBMC inhibition capacities in MLR assays (day 1: 3 ± 4 %; day 2: 10 ± 7 %; (Table 4). Indeed, 3 days of culture were necessary to restore a full and stable PBMC inhibition ability (day 3: 40 ± 9 %; day 4: 42 ± 2 %; day 5: 43 ± 1 %) (Table 4; Fig. 5).

**Table 4:** MSC thawing: pre and post-thaw MSC immunosuppressive properties

	(%)	Min.	Max.	Mean ± SD
Day 0	Recovery	61	84	72.8 ± 10.4
	Viability	61	71	65.1 ± 4.4
	Immunosuppression	5	5	5.0 ± 0.0
Day 1	Recovery	21	54	41.8 ± 12.9
	Viability	86	91	88.8 ± 2.1
	Immunosuppression	0	10	2.9 ± 4.1
Day 2	Recovery	19	40	30.3 ± 11.3
	Viability	78	87	83.9 ± 4.0
	Immunosuppression	4	19	10.3 ± 7.4
Day 3	Recovery	21	36	26.8 ± 10.8
	Viability	74	97	85.5 ± 16.3
	Immunosuppression	34	47	40.5 ± 8.6
Day 4	Recovery	21	34	28.3 ± 6.5
	Viability	89	93	90.3 ± 2.3
	Immunosuppression	40	43	41.8 ± 2.2
Day 5	Recovery	31	42	36.3 ± 7.5
	Viability	85	91	88.0 ± 4.2
	Immunosuppression	42	44	42.6 ± 1.3

Results are shown for MSC recovery, viability and immunosuppressive properties (%) at different times after thawing and replating in T-flasks with complete media

Recoveries were very low until day 4 (Fig. 5) before a slight increase thereafter indicating that cells had begun to re-proliferate. Thus, restoration of MSC immunosuppressive properties correlated with a stop in MSC loss. Nevertheless, cells did not require re-culture to maintain their differentiation capacities towards adipocytic, osteoblastic and chondroblastic lineages.

## Discussion

The purpose of this paper was to demonstrate the feasibility and describe the difficulties of setting up a large MSC bank from allogeneic donors for use in academic clinical trials. The constitution of a human third-party MSC bank from screened healthy volunteer donors and the follow-up of different aspects of this activity since 2007 are developed. Results from 68 large scale clinical expansions are described and discussed. Methods of generating clinical-grade MSCs have already been described by other groups but, to our knowledge, this is the first paper describing such a large and detailed banking experience [14, 15].

The absence of co-stimulatory molecules and human leucocyte antigen (HLA) class II molecules, as well as low HLA class I expression on MSCs, make them ideal for allogeneic use. MSC produced and used in our clinical trials are of allogeneic origin but do not seem to elicit immune responses. Indeed, HLA-mismatched MSC manufactured by the same process were shown to be weakly immunogenic after infusion into HSCT recipients [16]. One concern when expanding large number of MSC is the number of passages required to meet dose requirements. Some studies indicate that MSC properties change with passages and that immunosuppressive capacities become less potent when multiplying passages [17, 18]. Von Bar et al. published recently clinical results that suggest that acute GVHD patients treated with early passage MSC had a better survival than those treated with late passage cells [19]. In our manufacturing process, MSC are only cultured for three passages (14 days of primary culture and 2 passages of 7 days each). This is quite low and comparable to a MSC culture process yielding comparable cell numbers in four shorter passages as reported by Sabatino et al. [14]. However, it is now generally accepted that MSC will begin to senesce after a certain number of cell divisions and that this is best evaluated by their population doubling level (PDL) rather than by the number of passages or the duration of culture. As it is very difficult to evaluate the starting number of MSC in the initial culture (mixture of mononuclear cells), most labs start counting MSC cumulative population doubling at the end of the primary culture (first passage). The calculated PDL of our MSC culture ranged from 2.38 to 7.18 with a median of 4.69. This is consistent with standard MSC protocols showing 2.5–3 population doublings per passage. Moreover, the observed PDL are still below the PDL correlating with occurrence of MSC senescence (PDL from 10 to 40). However, the PDL of these cells in vivo before collection of the BM, which also depends on donor age, are not known. During our large culture experience, we were able to produce 1 to 45 doses of MSCs from each BM sample in only 3 passages demonstrating the efficacy of the process. We consider that it is very important to produce MSC from multiple donors for two main reasons: First, to limit the number of passages and second to have a large variability of donors. Indeed, it has been suggested that if one donor is used to produce a multiplicity of MSC doses to treat a lot of patients, a potency bias may be observed [20]. Indeed, MSC from different donors may have different interferon-gamma responsiveness (low or high IDO inducers) and thus different potency. The outcome of a patient receiving MSC from a low or high responder may be different. On the other hand, cells that are subject to a high proliferative pressure and late passage random donor MSC could be less effective than early passage MSC [19]. These factors may explain the below expectation results of the phase III trial of random donor MSC in steroid-resistant GVHD with the Prochymal^R^ industrial MSC product [20, 21]. Indeed, despite promising phase II trials, this phase III clinical trial did not meet its primary end point [22]. However, the product has been approved in two countries based on statistically significant benefits observed at certain disease sites.

Despite low passage cultures, we have been able to produce 464 MSC doses in 68 cultures that allowed us to create an allogeneic “off-the-shelf” MSC bank with a large number of frozen aliquots.

Up to June 2015, 290 MSC bags have been released and safely infused to patients included in our 6 current clinical protocols. We have evaluated cell viability and recovery from these thawed MSC products. Viability of fresh cells (90.3 ± 4.3 %) dropped to 76 ± 9.1 % after thawing (mean loss 14 %), but remained sufficient for meeting release criteria. These results compare favorably with those of Polchow et al. who describe a 20 % loss of viability (from 84.4 ± 9.4 to 67.4 ± 7.6 %) with thawing after short-time cryopreservation of human umbilical cord artery-derived cells (HUCAC) [23]. However, this difference can be explained by the fact that these cells were of different origin and cryopreserved in vials and not in bags. This was also the case for other studies that are difficult to compare because of dysparities in origin or culture stage of cells, cryopreservation and thawing methods [24–26].

Beside viability and recovery, freezing/thawing steps may also affect MSC potency. Indeed Galipeau et al. reported recently that post-thaw cells have impaired immunosuppressive properties [27]. They demonstrate that post-thawed MSC up-regulate heat-shock proteins, are refractory to interferon–γ-induced up-regulation of indoleamine 2,3-dioxygenase (IDO) and are compromised in suppressing CD3/CD28/-driven T cell proliferation. These properties were fully restored following 24 h of post-thaw MSC culture. In order to assess whether potency of our MSC presented similar kinetics, we thawed MSC bags and seeded cells back in culture for different periods of time before evaluating their viability, proliferation and potency (immunosuppressive properties and differentiation abilities). In accordance with Galipeau et al., we observed that after thawing, MSC are compromised in suppressing CD3/CD28/-driven T-cell proliferation. In our hands, 3 days of culture were necessary to fully restore stable immunosuppressive properties. This correlated with a recovery of their proliferative potential, indicating that MSC probably need a 3-day recovery period after thawing. However, differentiation potential was maintained even when evaluated immediately after thawing. Nevertheless, the relevance of these in vitro findings for the immunosuppressive efficacy of MSC is unknown. In vivo recovery of various cell properties may be quite different in the recipient body environment. Indeed, Von Bahr et al. have demonstrated that in vitro testing of MSC inhibitory capacity in MLC or mitogenic response to PHA did not correspond with in vivo responses [19]. MSC potency defined by MLR assay may not be a good predictor of their in vivo effects. Krampera et al. recently published a working proposal of the ISCT for immunological characterization of MSC [28]. It mentioned the urgent need as part of cell manufacturing to develop and implement robust potency assays that may be associated with clinical effects and suggest different proposals including the use of IFN-γ and/or TNF-α in vitro primed MSC; investigation of cell-surface markers expression by flow cytometry for characterizing MSC immunological properties; the use of purified immune effector cells subsets in MLR instead of unselected peripheral blood mononuclear cells (PBMC), the of study some suppressor pathways induced by MSC such as IDO activation and the use of animal models to evaluate MSC immunosuppressive properties The aim of standardization is to obtain reproducible and consistent in vitro data that reflect immunological properties of MSCs infused to patients.

In this paper, we report optimization of clinical-grade large-scale MSC expansion with the set-up of a MSC bank and summarize 8 years of experience. The technical process in place leads to per donor cell yields that are sufficient for therapeutic purposes (median of 546 × 10E6 MSC/culture = 4 doses of 2 × 10E6 cells/kg for an average 75-kg patient). However, the manipulation of large quantities of T-flasks is a hurdle (time consuming, space consuming and open system) and, to avoid these disadvantages, we have validated the fully automated closed Quantum^®^ bioreactor for our process and published our observations [29]. The major advantages of the bioreactor were that (1) cells grow better in the Quantum^®^ than in flasks, (2) working time is shorter especially at the final harvest step, and (3) all the feeding tasks are done automatically. This system thus allows production of large quantities of MSC. However, the cost of the device counterbalances its advantages, so that we did not implement it in our manufacturing strategy.

In the meantime, MSC have become considered as advanced therapy medicinal products (ATMP) by the European Medicines Agency (EMA), are under European Regulation N° 1394/2007 and must be produced in compliance with Good Manufacturing Practices (GMP). GMP requires thorough analysis of critical aspects such as donor screening, production processes and quality controls of expanded MSC (safety, identity and efficacy) [30]. Our MSC are produced under strict and defined parameters from donor screening to culture reagents and processes, using screened and irradiated FBS batches and subjected to extensive quality controls for safety (bacteriological analysis, endotoxin and mycoplasma detection), identity (FACS analysis according to ISCT criteria) and potency (immunosuppressive properties). Due to extensive washing and dilution of the cells before cryopreservation, residual reagents (trypsin–EDTA, FBS, and penicillin/streptomycin) are present at extremely low levels (0.00138, 0.00024 and 0.000000286 %, respectively) after thawing. In addition, karyotype analysis performed on each batch has consistently shown genetic stability of the cells. We also showed that despite cell trapping in the lungs after intravenous infusion, this did not result in deleterious effects on lung function ([31]. In an attempt to improve aseptic conditions, GMP recommends the development of closed processes. For that purpose, we have successfully implemented in 2012 a new method for selection of mononuclear cells from BM samples with the fully closed Sepax^R^ device [32]. Similarly, as explained earlier, we have validated the Quantum^R^ bioreactor but didn’t implement it in our manufacturing process because of cost concerns. However, closed systems are not an absolute requirement of GMP and several groups have described GMP-compliant culture processes in Cellstacks [33] or T-flasks [15]. We are now in the last phase of GMP implementation in our process of MSC banking. Briefly, major changes applied to the process concern the environment (extensive monitoring, class B room with adapted controls of work environment and equipment, clothes, hygienic and safety rules, sanitization of the clean room and maintenance/checking of equipment), technical process (replacement of some reagents, use of cellstacks instead of T-Flasks, separation of people involved in production and in quality control), quality control (formal validation of all internal QC methods and use of GMP-certified laboratories for subcontracted analyses, quarantine status before release of batches by a qualified person), reagents (quarantine status until release by the qualified person according to their specifications) and a considerable extension of the documentation system and traceability of all processes.

## Conclusion

In conclusion, we report here an efficient clinical-grade MSC banking activity in place for more than 7 years. This activity now has to be performed in accordance with GMP requirements. A significant challenge remains the development and implementation of standardized potency assays and the demonstration of a correlation with relevant endpoints in clinical trials.

## Abbreviations

MSC: mesenchymal stem/stromal cells
HLA: human leukocyte antigen
GVHD: graft-versus-host disease
CD: Crohn’s disease
RA: rheumatoid arthritis
ISCT: International Society of Cellular Therapy
LTCG: Laboratory of Cell and Gene Therapy
BM: bone marrow
GVHD: graft versus host disease
NMAHCT: non myeloablative hematopoietic cell transplantation
GVT: graft versus tumor
EBMT: European Group for Blood and Marrow Transplantation
HCT: hematopoietic cell transplantation
HSC: hematopoietic stem cells
CBT: cord blood transplantation
GMP: good manufacturing practise
UMN: urgent medical need
SOP: standard operating procedures
MNC: mononuclear cells
PBS: phosphate-buffer saline
DMEM: Dulbecco’s modified eagles medium
EDTA: ethylène diamine tetra acétate
FBS: foetal bovine serum
P/S: penicilline/streptomycine
HAS: human serum albumin
DMSO: diméthylsulfoxyde
CFU-F: colony forming unit fibroblasts
MLR: mixed lymphocyte reaction
R&D: research and development
PBMC: peripheral blood mononuclear cells
HSCT: human stem cell transplantation
IDO: indoléamine oxidase
HUCAC: human umbilical cord artery derived cells
CPA: cryoprotectant
EMA: European medicines agency
ATMP: advanced therapeutic medicinal product
